# Contraceptive-Induced Weight Gain—Myth and Reality Review

**DOI:** 10.3390/life16040553

**Published:** 2026-03-27

**Authors:** Tudor Butureanu, Ana-Maria Apetrei, Raluca Anca Balan, Ana-Maria Haliciu, Ioana Pavaleanu, Demetra Socolov, Razvan Socolov

**Affiliations:** 1 Grigore T Popa University of Medicine and Pharmacy, 700115 Iasi, Romania; tudorandreib@gmail.com (T.B.); socolov.razvan@gmail.com (R.S.); 2 Department of Mother and Child, “Elena Doamna” University Hospital of Obstetrics and Gynecology, 700398 Iasi, Romania; 3 Pathology Department, “Elena Doamna” University Hospital of Obstetrics and Gynecology, 700398 Iasi, Romania; 4 Department of Mother and Child, “Cuza Vodă” University Hospital of Obstetrics and Gynecology, 700036 Iasi, Romania

**Keywords:** contraception, fat, weight-gain, progesterone, estrogen, hormonal, endometriosis, polycystic ovary syndrome

## Abstract

The perception that hormonal contraception causes weight gain is a general belief that frequently hinders the initiation and continuation of effective family planning. This narrative review analyses data from Cochrane systematic reviews and recent pharmacogenomic studies to separate patient perception from metabolic reality. Analysis of high-quality data, including Cochrane systematic reviews, indicates that the association between Combined Hormonal Contraceptives (CHCs)—including oral pills, the transdermal patch, and the vaginal ring—and weight gain is not supported by consistent high-quality evidence. Placebo-controlled trials demonstrate that these methods are weight-neutral on average. Perceived weight increases in CHC users are likely mediated in part by fluid retention linked to the estrogenic stimulation of the Renin–Angiotensin–Aldosterone System (RAAS), rather than adipose tissue accumulation. Conversely, Depot Medroxyprogesterone Acetate (DMPA) represents a verified clinical risk for weight gain, showing a demonstrated clinical association with significant fat mass accumulation. Hypothesized biological mechanisms for this increase include hypothalamic appetite stimulation and glucocorticoid-like activity. The etonogestrel implant occupies a complex middle ground. While population-level data suggests weight neutrality, recent exploratory pharmacogenomic research has identified a specific variant in the Estrogen Receptor 1 (ESR1) gene. For the minority of women carrying this variant, the implant may trigger clinically significant weight gain, suggesting a biological basis for their subjective experience despite statistical evidence. Ultimately, the persistence of the weight gain concern is fueled by the nocebo effect and the misattribution of natural age-related weight trajectories to contraceptive use.

## 1. Introduction

The belief that hormonal contraception causes weight gain is the primary reason women delay starting effective family planning or stop it early. This perception, pervasive among patients and clinicians alike, acts as a formidable barrier to the initiation of effective family planning and serves as a primary driver for early discontinuation. The narrative that the pill can make you fat is not merely a cosmetic concern; it is a public health challenge that significantly influences contraceptive prevalence rates, unintended pregnancy statistics, and the overall reproductive autonomy of women globally and other disease treatment compliance, like endometriosis or ovarian polycystic syndrome. We aim to deconstruct this pervasive myth through a rigorous analysis of pharmacological, physiological, and epidemiological evidence, distinguishing between the reality of the patient experience and the statistical truth of randomized controlled trials.

The persistence of the weight gain myth is rooted in a complex interplay of historical, psychological, and biological factors. Historically, the earliest oral contraceptive formulations contained high doses of estrogen (up to 150 µg of mestranol or ethinyl estradiol) and first-generation progestins, which indeed caused significant fluid retention and anabolic side effects [1]. While modern formulations have dramatically reduced hormonal dosages—often containing as little as 20 µg of ethinyl estradiol—the reputational legacy of their predecessors endures. This historical context is compounded by the nocebo effect, where the anticipation of a negative side effect precipitates its perception or even its somatic realization, a phenomenon well-documented in placebo-controlled contraceptive trials [2,3,4].

The demographic typically initiating contraception—adolescents and young adults—coincides with a life stage characterized by natural physiological growth and metabolic shifts. Epidemiological data indicates that the average American woman gains approximately 12 pounds per decade during early adulthood regardless of contraceptive use [5]. The conflation of this natural age-related weight trajectory with the initiation of hormonal therapy creates a potent confirmation bias. When a woman starts a contraceptive method and subsequently gains weight due to lifestyle changes or aging, the contraceptive is almost invariably identified as the causal agent.

This study provides an exhaustive evaluation of the evidence across all major classes of hormonal contraception: Combined Hormonal Contraceptives (CHCs), Progestin-Only Pills (POPs), Long-Acting Reversible Contraceptives (LARCs) including implants and intrauterine systems, and injectable depot medroxyprogesterone acetate (DMPA). By synthesizing data from Cochrane systematic reviews, metabolic studies, and emerging pharmacogenomic research, we aim to provide a nuanced, evidence-based framework that moves beyond the binary debate of myth vs. fact to a personalized understanding of individual metabolic responses.

## 2. Methods

Search Strategy and Data Sources This narrative review relied on existing high-quality evidence, prioritizing data from the Cochrane Collaboration, Embase, Scopus, and PubMed. The search strategy focused on identifying literature that addressed the association between hormonal contraception and weight modulation across three domains: clinical outcomes, physiological mechanisms, and pharmacogenomics.

Primary data sources included:

- **Cochrane Systematic Reviews**: Utilized as the gold standard for evaluating causal links in large populations, specifically examining randomized controlled trials (RCTs) of combination contraceptives and progestin-only methods.
- **Pharmacogenomic and Metabolic Studies**: Targeted searches were conducted for recent studies investigating genetic variants (e.g., ESR1) and metabolic pathways (e.g., RAAS, insulin sensitivity) to address individual variability in weight response.
- **Neuroimaging and Behavioral Research**: Evidence regarding hypothalamic appetite regulation and neural reward processing was included to evaluate mechanistic differences between contraceptive methods, particularly for injectable medroxyprogesterone acetate.

**Inclusion and Exclusion Criteria**

To ensure a rigorous analysis of the myth vs. reality we applied the following criteria:

- **Population:** Adolescents and women of reproductive age initiating or maintaining hormonal contraception were included in the study only, with women outside these categories not being considered.
- **Interventions:** All major classes of hormonal contraception were evaluated, including Combined Hormonal Contraceptives (oral pills, transdermal patch, vaginal ring), Progestin-Only Pills (POPs), Long-Acting Reversible Contraceptives (implants, hormonal IUDs), and Depot Medroxyprogesterone Acetate (DMPA).
- **Comparators:** Preference was given to studies utilizing placebo controls, non-hormonal controls (e.g., Copper IUD), or active comparator designs to distinguish pharmacological effects from secular weight trends.
- **Outcomes:** The primary outcome of interest was quantitative weight change (kg). Secondary outcomes included changes in body composition (fat mass vs. lean mass vs. total body water), discontinuation rates due to weight, and specific adverse effects such as fluid retention or appetite changes. Studies not involving hormonal contraceptives, without weight-related outcomes, studies where contraceptives were used for non-contraceptive therapeutic purposes only, and articles without primary or synthesized scientific evidence, as well as animal or laboratory studies were excluded from our study.

**Data Synthesis and Analysis:** Data were synthesized narratively to provide a nuanced framework that moves beyond binary conclusions. The analysis was stratified by contraceptive delivery method to isolate distinct physiological mechanisms:

1. **Fluid vs. Fat:** Differentiating weight gain caused by estrogen-mediated fluid retention (RAAS stimulation) from true adipose tissue accumulation.
2. **Population Mean vs. Individual Variance:** Contrasting population-level statistical averages with data on genetic susceptibility and outliers to validate patient experiences that deviate from the mean.
3. **Behavioral attribution:** Evaluating the role of the nocebo effect and age-related metabolic shifts natural age-related metabolic and lifestyle transitions in the perception of weight gain.

## 3. Physiological Mechanisms of Weight Modulation

To evaluate the clinical data, we must first understand the theoretical biological plausibility of hormone-induced weight changes. The interaction between exogenous sex steroids and body composition is mediated through three primary pathways: fluid regulation via the Renin–Angiotensin–Aldosterone System (RAAS), hypothalamic appetite regulation, and modulation of adipocyte metabolism.

### 3.1. Estrogen and the Renin–Angiotensin–Aldosterone System (RAAS)

The most common cause of short-term weight fluctuation in women initiating combined hormonal contraception is fluid retention, often misidentified as adipose gain. Ethinyl estradiol, the estrogenic component of most combined methods, exerts a potent influence on the hepatic synthesis of serum proteins, including angiotensinogen [6]. Angiotensinogen is the substrate for renin, the rate-limiting enzyme in the RAAS cascade. Elevated levels of angiotensinogen lead to increased production of Angiotensin I, which is converted by Angiotensin-Converting Enzyme (ACE) to Angiotensin II. Angiotensin II is a powerful vasoconstrictor that stimulates the adrenal cortex to secrete aldosterone. Aldosterone acts on the distal tubules of the nephron to increase sodium reabsorption; water passively follows sodium to maintain osmotic balance, resulting in an expansion of extracellular fluid volume [6].

In a natural ovulatory cycle, the endogenous progesterone produced during the luteal phase possesses a high affinity for the mineralocorticoid receptor, where it acts as an antagonist. This competitive inhibition blocks the action of aldosterone, promoting natriuresis (sodium excretion) and preventing excessive fluid retention despite high estrogen levels [7,8,9]. However, many synthetic progestins used in older contraceptive formulations (e.g., levonorgestrel, norethindrone) lack this specific anti-mineralocorticoid activity. Consequently, the estrogen-driven increase in aldosterone goes unopposed, leading to measurable fluid retention and the sensation of bloating or swelling that patients interpret as weight gain [7].

Recognizing this mechanism, pharmaceutical development introduced fourth-generation progestins like drospirenone. Chemically derived from spironolactone, a potassium-sparing diuretic, drospirenone exhibits potent anti-mineralocorticoid activity [10,11]. It effectively counters the estrogenic stimulation of the RAAS, neutralizing fluid retention. This mechanistic distinction explains why clinical trials comparing drospirenone-containing COCs to older formulations often show a slight weight loss or greater weight stability in the drospirenone arm [1].

### 3.2. Progestins and Hypothalamic Appetite Regulation

While estrogen is primarily linked to fluid status, progestins are scrutinized for their potential impact on energy balance and appetite. The hypothalamus serves as the central integrator of energy homeostasis, receiving signals from peripheral hormones such as ghrelin (orexigenic/hunger-promoting) and leptin (anorexigenic/satiety-promoting) [12,13]. Progesterone receptors are densely expressed in hypothalamic nuclei involved in feeding behavior. Theoretical models suggest that progestins may exert a glucocorticoid-like effect. Medroxyprogesterone acetate (MPA), the active agent in the injectable depot, binds with significant affinity to the glucocorticoid receptor [14]. Glucocorticoids are known to stimulate appetite, particularly for calorie-dense foods, and to promote the deposition of visceral adipose tissue [15].

Neuroimaging studies using functional MRI have suggested compelling evidence for this pathway in DMPA users. Researchers at the University of Southern California found that women using DMPA showed increased neural activity in the brain’s reward centers when viewing images of high-calorie foods compared to non-users [16]. This suggests that for susceptible individuals, certain potent progestins may fundamentally alter the neural response to food cues, overriding homeostatic satiety signals and driving hyperphagia.

Conversely, estrogen typically acts as an anorexigenic signal in animal models, reducing food intake and increasing energy expenditure. The balance between the appetite-suppressing effects of estrogen and the potentially appetite-stimulating effects of progestins determines the net metabolic impact of a combined contraceptive [17]. This delicate balance may explain the variability in individual responses; a woman with a genetic predisposition to progestin sensitivity may experience increased hunger, while another may find her appetite unchanged or diminished.

### 3.3. Adipocyte Metabolism and Insulin Sensitivity

Beyond the central nervous system, sex steroids interact directly with adipose tissue. Adipocytes are not merely storage vessels but active endocrine organs. Some studies have suggested that progestins can stimulate lipoprotein lipase (LPL) activity, the enzyme responsible for hydrolyzing triglycerides into free fatty acids for uptake by fat cells [18]. An upregulation of LPL, particularly in femoral-gluteal fat depots, could theoretically facilitate fat storage. However, the clinical data regarding insulin sensitivity is mixed. While pregnancy (a high progesterone state) induces physiological insulin resistance, modern low-dose contraceptives generally have a negligible impact on carbohydrate metabolism in healthy women [14]. In women with existing metabolic derangements, such as Polycystic Ovary Syndrome (PCOS) or obesity, the impact may be more nuanced. Some evidence indicates that androgenic progestins (like levonorgestrel) might slightly decrease glucose tolerance, potentially influencing weight regulation, whereas non-androgenic progestins do not [11].

### 3.4. Anabolic Effects and Lean Body Mass

A frequently overlooked aspect of weight change is the composition of the mass gained. Weight gain on the scale is not synonymous with fat gain. Androgenic progestins, derived from 19-nortestosterone, possess residual androgenic activity. This anabolic potential could, in theory, promote the retention of lean muscle mass [19]. Studies utilizing dual-energy X-ray absorptiometry (DEXA) or bioelectrical impedance have occasionally noted increases in lean body mass in users of hormonal IUDs and implants, distinct from fat mass accumulation [20]. This finding challenges the “fat gain” narrative, suggesting that some “weight” gain may be metabolically healthy muscle tissue or bone density preservation, particularly in younger users. Table 1 presents a comparative analysis of fat and lean mass changes, according to the type of hormonal contraceptive device.

## 4. Combined Hormonal Contraceptives (CHCs): Evidence from Systematic Reviews

Combined Hormonal Contraceptives (CHCs), encompassing oral pills, the transdermal patch, and the vaginal ring, represent the most widely used class of reversible contraception. Due to their ubiquity, they have been the subject of the most rigorous scrutiny regarding side effects. Comparative weight outcomes in CHCs are presented in Table 2.

### 4.1. Combined Oral Contraceptives (COCs)

The Cochrane Collaboration, recognized globally for its high standards of evidence synthesis, has produced definitive reviews on the association between COCs and weight. The most recent major update analyzed 49 randomized controlled trials (RCTs) to determine if a causal link exists [1]. The overarching conclusion of this massive data synthesis is that the available evidence is insufficient to determine a causal effect of combination contraceptives on weight, but crucially, no large effect was evident. In studies where women were randomized to either a COC or an inert placebo pill, weight changes were strikingly similar between groups. For example, trials have shown that while women in the active treatment group might gain an average of 0.5 to 1 kg over a year, the placebo group often gained a comparable amount [21]. This demonstrates that the weight gain observed in clinical practice is largely attributable to the natural trajectory of weight gain over time (secular trend) rather than the pharmacological action of the contraceptive. A cross-sectional observational study found that users of combined oral contraceptives (COCs) reported more weight gain and mood-related side effects than users of other contraceptive methods. Satisfaction differed across methods, emphasizing the importance of individualized counseling to address patient concerns and support informed contraceptive choices [22].

#### 4.1.1. Comparisons of Progestin Types

We also compared different formulations to assess if specific progestins were more weight-friendly.

- Levonorgestrel vs. Desogestrel: Comparisons between these second and third-generation progestins generally showed no substantial difference in weight outcomes or discontinuation rates due to weight [1].
- Drospirenone: As noted in the mechanistic section, drospirenone-containing pills (e.g., Yaz, Yasmin) have demonstrated a distinct profile. In one analysis, the odds ratio for losing more than 2 kg was 9.22 (95% CI 1.79 to 55.04) for women on drospirenone compared to levonorgestrel [1,23]. This statistically significant finding supports the hypothesis that drospirenone’s anti-mineralocorticoid activity reduces water retention, leading to a slight reduction in weight or prevention of fluid-related gain. However, this should not be interpreted as fat loss; rather, it is the absence of fluid retention.

#### 4.1.2. Discontinuation Due to Weight

A critical metric in these trials is the discontinuation rate. If weight gain were a severe and frequent side effect, one would expect significantly higher dropout rates in the active treatment arms compared to placebo. The Cochrane review found that discontinuation due to weight change did not differ significantly between groups [1]. Another study concluded that eating-disordered symptoms and perceived weight changes were associated with an increased likelihood of OC discontinuation among young women [24]. This suggests that while women may perceive weight gain, it is often not severe enough or distinct enough from their baseline fluctuations to prompt discontinuation in a blinded trial setting where the nocebo effect is controlled.

### 4.2. The Transdermal Patch

The contraceptive patch (e.g., Evra/Ortho Evra) delivers norelgestromin and ethinyl estradiol transdermally, bypassing first-pass hepatic metabolism. This route theoretically provides more stable serum hormone levels, avoiding the daily oral administration. Clinical trials evaluating the patch have largely mirrored the findings for COCs. A pooled analysis of phase III clinical trials found that the mean increase in body weight from baseline to the end of treatment was only 0.3 kg [11]. Furthermore, nearly 80% of participants remained within 5% of their baseline weight.

A detailed Italian study utilizing bioelectrical impedance analysis (BIA) provided granular insight into the composition of this weight change. Over a 6-month period, patch users gained a mean of 0.64 kg. However, BIA revealed that Total Body Water (TBW) increased by 0.51 L [25,26]. The study authors concluded that the minimal weight gain was almost entirely attributable to “adaptive interstitial gel hydration”—essentially, a mild physiologic fluid shift—rather than an increase in adipose tissue. Importantly, this fluid shift did not correlate with increases in blood pressure, indicating it was a benign physiologic adaptation [25,26].

Despite these reassuring biological data, the patch is often subject to higher rates of discontinuation due to perceived side effects. This highlights the disconnect between the measured physiological reality (0.6 kg of fluid) and the patient’s subjective experience of feeling swollen.

### 4.3. The Vaginal Ring

The vaginal ring (e.g., NuvaRing) releases etonogestrel and ethinyl estradiol directly into the vaginal epithelium. Like the patch, it offers steady-state hormone release and lower systemic ethinyl estradiol exposure (15 µg/day equivalent) compared to many oral pills [27,28]. Comparative studies have consistently shown the ring to be weight-neutral. In a multicenter trial comparing the ring to a combined oral contraceptive, ring users demonstrated no evidence of weight gain compared to non-users over a one-year period [27,28]. In fact, some studies have noted that ring users report high satisfaction regarding weight stability, possibly due to the lower estrogen dose minimizing the RAAS-mediated fluid retention described earlier.

A specific study assessing body image and sexual function in ring users found that while there was a statistically significant but slight increase in BMI (from 21.9 to 22.1) over 6 months, this change was clinically negligible and did not negatively impact body image perception or sexual function. A study evaluating body image and sexual function among vaginal ring users reported a small but statistically significant increase in BMI (from 21.9 to 22.1) over six months. However, this change was clinically negligible and did not adversely affect body image perception or sexual function [29]. The stability of weight on the ring makes it an attractive option for women specifically concerned about estrogen-related bloating.

## 5. Progestin-Only Contraceptives (POCs)

Progestin-only methods are essential for women who have contraindications to estrogen (e.g., migraine with aura, hypertension, history of VTE). As they lack the estrogen component, the mechanism of fluid retention via RAAS is largely absent. However, the metabolic effects of the progestin itself become the primary focus.

### 5.1. Progestin-Only Pills (POPs)

This pill category includes traditional formulations (norethindrone) and newer options (desogestrel, drospirenone). A Cochrane review of 22 studies on POCs found limited evidence of weight change. Mean weight gain across most studies was less than 2 kg over 6 to 12 months [23]. When studies followed women for longer periods (2–4 years), weight gain did increase, often doubling compared to the 1-year mark. However, this gain was observed equally in control groups using non-hormonal methods [23]. This observation strongly supports the age-related weight gain hypothesis: the weight gain is a function of time and aging, not the drug. The lack of a statistically significant difference between POP users and non-users confirms that progestin-only pills, in aggregate, are weight-neutral.

### 5.2. The Etonogestrel Implant (Nexplanon/Implanon)

The contraceptive implant is a highly effective Long-Acting Reversible Contraceptive (LARC). It has a complex reputation: while generally considered weight-neutral in population studies, it is frequently cited by patients as a cause of rapid, severe weight gain. In large comparative trials, such as those comparing implant users to copper IUD users (the ultimate non-hormonal control), no significant difference in weight gain is typically found after adjusting for confounders [20]. For instance, one prospective cohort study observed a mean weight increase of only 0.1 kg in implant users after 12 months, which was actually lower than the gain observed in LNG-IUS (0.5 kg) and Copper IUD (0.4 kg) users [20]. Another study found a mean gain of 3.2 kg over 27 months, which aligns with the expected secular weight gain trend for young adult women [30].

### 5.3. The Genetic: ESR1 Variants

The discrepancy between the average weight neutrality and the individual severe gain was recently illuminated by a landmark pharmacogenomic study from the University of Colorado [30]. Researchers hypothesized that genetic variations in hormone metabolism might explain why some women gain weight while most do not.

They genotyped 276 implant users and identified a specific variant in the Estrogen Receptor 1 (ESR1) gene (rs9340799). The findings were stark:

- Wild-Type/Heterozygous: Women with normal variants gained an average of ~3–4 kg over the study period.
- Homozygous Variant: Women with two copies of the risk variant gained an average of 14.1 kg more than their counterparts [30].

This finding is transformative. It validates the myth as a biological reality for a specific genetic sub-population (estimated at roughly 5–10% of the cohort). It suggests that for these women, the interaction between the etonogestrel released by the implant and their specific estrogen receptor configuration triggers a profound metabolic shift, likely involving appetite regulation or adipogenesis.

In the same context, another study showed that genetic variations can influence etonogestrel levels in contraceptive implant users. These findings highlight the potential role of pharmacogenetics in individualizing hormonal contraceptive efficacy and safety [31].

### 5.4. The Levonorgestrel Intrauterine System (LNG-IUS)

The hormonal IUD (e.g., Mirena, Kyleena) releases levonorgestrel directly into the uterine cavity. Systemic absorption is minimal compared to oral or injectable routes. Consequently, systemic side effects are expected to be lower.

Systematic reviews and comparative trials consistently show that the LNG-IUS is weight-neutral. In direct comparisons with the copper IUD, weight changes are statistically indistinguishable [20]. A detailed body composition study found that while LNG-IUS users gained a small amount of weight (0.5 kg/year), they also experienced an increase in lean body mass [20]. Furthermore, cognitive measures of appetite (cognitive restraint, disinhibition) actually decreased in LNG-IUS users, contradicting the notion that progestins universally stimulate hunger [20]. Despite this, surveys indicate that women often believe the hormonal IUD causes more weight gain than the copper IUD [23]. This perception gap may be driven by the amenorrhea associated with the LNG-IUS, which women may psychologically associate with backing up or retaining impurities, leading to a psychosomatic sensation of heaviness.

## 6. Depot Medroxyprogesterone Acetate (DMPA)

Among all modern hormonal contraceptives, Depot Medroxyprogesterone Acetate (DMPA), commonly known as “the shot” stands apart as the only method with consistent, high-quality evidence linking it to significant weight gain and fat mass accumulation. Unlike the neutral or minimal gains seen with pills and implants, DMPA use is associated with substantial weight increases in a significant proportion of users. Longitudinal comparisons reveal that DMPA users can gain between 5.1 kg and 6.6 kg over periods ranging from 3 to 10 years, significantly more than users of other hormonal or non-hormonal methods [23,32]. A pivotal study analyzing body composition changes over 36 months found that DMPA users gained significantly more total weight (+5.1 kg) compared to oral contraceptive users and non-hormonal controls. More concerning was the composition of this gain: DMPA users experienced a +4.1 kg increase in body fat mass and a statistically significant increase in the central-to-peripheral fat ratio [33]. This indicates a specific accumulation of visceral adipose tissue, which is metabolically distinct from subcutaneous fat and carries higher risks for insulin resistance and cardiovascular disease. Another recent longitudinal study revealed that injectable progestin contraceptives may contribute to measurable weight gain over time, finding a significant increase in body weight and BMI after 12 months of DMPA use, with mean weight change of +0.9 kg after 12 months [34].

### 6.1. Mechanisms of DMPA-Induced Weight Gain

The mechanism driving this gain is multifaceted and distinct from the fluid retention seen with COCs. The assessment of weight gain risk across different contraceptive methods is presented in Table 3.

1. Hypothalamic Dysregulation: DMPA appears to alter the brain’s reward processing. The University of Southern California neuroimaging study hypothesized that DMPA users had heightened activation in the orbitofrontal cortex and striatum—regions governing reward and motivation—when presented with high-calorie food cues [16]. This neural hyper-responsiveness manifests as increased appetite and cravings.
2. Glucocorticoid Activity: MPA binds to the glucocorticoid receptor with an affinity that rivals cortisol. Chronic glucocorticoid receptor activation promotes adipogenesis (creation of fat cells), particularly in the visceral abdominal depot [14].
3. Insulin Resistance: Some data suggest DMPA may induce a mild state of insulin resistance, shifting metabolism toward fat storage rather than oxidation, although this effect is variable [35].

Not all DMPA users gain weight. The response appears to be bimodal: some gain significantly, while others maintain stability. Research has identified a powerful predictive tool for clinicians: early weight gain. Studies in adolescents found that those who gained more than 5% of their baseline body weight within the first 6 months of DMPA use were at extremely high risk for continued, excessive weight gain [36]. Conversely, those who did not gain significant weight in this initial window were unlikely to do so later. This 5% rule allows for a test-dose approach: initiate DMPA, monitor weight at the second injection (3 months) and third injection (6 months). If the trajectory is upward, immediate intervention or method switching is indicated to prevent long-term obesity. The data often obscures the experiences of specific demographic groups. Adolescents, racial minorities, and women with obesity may have distinct responses to hormonal contraception.

### 6.2. Growth vs. Gain in Adolescents

Adolescence is critical for bone density and body composition changes. Studies comparing adolescents on the implant versus the pill versus controls found that implant users had BMI changes similar to DMPA users, and higher than controls [37,38]. In this regard, a recent study found that both the subdermal progestin implant and combined oral contraceptives were generally well tolerated in adolescents with Type 1 Diabetes. The results support the safety of these contraceptive options in this population, though continued monitoring is recommended [39]. One study showed a mean BMI difference of +1.0 in implant users vs controls at 36 months. However, interpreting this is challenging due to the confounding factor of puberty. Adolescents are supposed to gain weight as they finish growing. The challenge lies in distinguishing healthy developmental weight gain from drug-induced adiposity. Additionally, the concern with DMPA in adolescents extends to bone mineral density (BMD). The hypo-estrogenic state induced by DMPA can impair peak bone mass acquisition. While this bone loss is generally reversible, the combination of potential weight gain and bone density issues requires careful counseling for teen users [14]. Moreover, the study by Pang et al. suggests that fluctuations in ovarian hormones may influence loss-of-control eating in adolescent girls. These findings highlight the potential role of hormonal changes in eating behaviors during adolescence [40].

### 6.3. Racial Disparities in Weight Response

Emerging evidence points to potential racial disparities in contraceptive-induced weight gain.

- Black Women and Weight Gain: Some studies have suggested that Black women may be more susceptible to weight gain on hormonal methods compared to White women. In a study of the implant, Black race was associated with a greater mean weight change across all contraceptive methods in crude analyses, although this significance sometimes attenuated in adjusted models [41]. Another study found that Black women were more than twice as likely to discontinue birth control due to concern about weight gain compared to White women [42].
- Genetic Context: The discovery of the ESR1 variant’s link to implant weight gain [31,33] raises the question of allele frequency. If the risk variant is more common in certain ancestral populations, it could explain the observed epidemiological disparities. Further research is urgently needed to map these pharmacogenomic markers across diverse populations to ensure equitable care.

### 6.4. Obesity and Contraception

The relationship between obesity and contraception is bidirectional.

1. Does Obesity Affect Efficacy? There is limited evidence that the transdermal patch may be less effective in women weighing >90 kg [43]. For emergency contraception, levonorgestrel (Plan B) shows reduced efficacy in women with higher BMI. However, for most daily or long-acting methods (Pills, IUDs, Implants), efficacy remains high regardless of BMI [43].
2. Do Contraceptives Worsen Obesity? The consensus from ACOG and RCOG guidelines is that the benefits of preventing pregnancy (which carries significant weight and metabolic risks) outweigh the risks of contraceptive-induced weight gain for obese women [44]. Hormonal methods do not appear to cause more weight gain in obese women compared to normal-weight women [45]. The primary concern is the additive risk of Venous Thromboembolism (VTE). Since obesity is an independent risk factor for VTE, and estrogen further increases this risk, the WHO and CDC Medical Eligibility Criteria often favor progestin-only methods (implants, IUDs) for women with severe obesity, not because of weight gain concerns, but because of safety profiles [43]. A large contraceptive study designed to assess whether contraceptive failure rates among users of COCs, patches, and vaginal rings are associated with increasing body mass index (BMI) concluded that overweight and obese women are not at a higher risk of contraceptive failure when using these methods [46]. Another review evaluating the effectiveness, safety, and metabolic considerations of contraceptive methods in women with obesity highlighted that most contraceptive options remain effective, but factors such as altered pharmacokinetics, cardiovascular risk, and counseling on long-acting reversible methods should be considered when selecting the most appropriate method [47].

## 7. The Myth: Nocebo, Attribution, and Body Image

To understand why the myth persists despite contrary evidence for most methods, we must look to psychology.

### 7.1. The Nocebo Effect

The nocebo effect is the phenomenon where negative expectations lead to negative outcomes. A striking study on the contraceptive implant illustrated this power. Women were randomized to receive an implant immediately or to a delay group. The implant group was counseled about potential side effects, including weight gain. At 3 months, significantly more women in the implant arm perceived weight gain compared to the control group. However, objective measurements showed no difference in actual weight between the groups [3]. The study authors concluded that the standard practice of counseling women to watch out for weight gain might be a self-fulfilling prophecy, priming patients to attribute normal fluctuations to the device and leading to unnecessary discontinuation. Another review highlights that concern about perceived weight gain can significantly influence women’s decisions when choosing hormonal contraception. The authors emphasize the importance of evidence-based counseling to address misconceptions and support informed contraceptive choices [21].

The initiation of contraception often aligns with major life transitions: starting college, entering a serious relationship, or moving in with a partner. These life stages are independently associated with lifestyle changes that promote weight gain (e.g., changes in diet, alcohol consumption, decreased physical activity).

- The Data: NHANES data confirms that the steepest trajectory of weight gain for US adults occurs in their 20 s and 30 s [5].
- The Error: A woman starts the pill at age 19. Over the next year, she gains 3 kg due to the natural age-related metabolic and lifestyle transitions phenomenon (lifestyle). She attributes the 3 kg entirely to the pill. This creates a powerful anecdotal narrative that no amount of placebo-controlled data can easily dismantle in the patient’s mind.

### 7.2. Discontinuation Dynamics

Perception drives behavior more than reality. Studies indicate that women who perceive weight gain are significantly more likely to discontinue their method, even if the scale shows they haven’t gained a single pound [24]. This creates a vicious cycle: specific counseling about weight gain increases fear -> fear leads to hyper-vigilance -> normal fluctuations are perceived as pathological -> the method is stopped -> the myth is reinforced in the user’s social circle.

## 8. Clinical Implications and Counseling Strategies

The divergence between the statistical average and the individual outlier requires a refined clinical approach. Providers must move away from dismissive language (“studies say it doesn’t happen”) toward validating, evidence-based counseling.

### 8.1. Evidence-Based Counseling Scripts

Instead of a blanket denial of weight gain, clinicians should use nuanced scripts:

- For COCs/Patch/Ring: “Large studies show that on average, women do not gain fat from these methods. However, some women experience fluid retention, which can feel like weight gain. This usually settles after 3 months. If you notice bloating, we can try a formulation with drospirenone to help with fluid balance.”
- For the Implant: “Most women stay weight neutral, but a small number of women have a genetic predisposition to gain weight. We can’t test for this gene yet, so we will monitor your weight. If you see a significant increase, we will believe you and we can remove it.”
- For DMPA: “The shot is the only method linked to appetite changes and weight gain in some women. We will weigh you at every injection. If you gain more than 5% of your body weight in the first 6 months, we should switch methods to prevent long-term gain.”

### 8.2. The Role of Precision Medicine

The identification of the exploratory ESR1 variant marks the beginning of precision contraception. In the future, a simple cheek swab could identify women at risk for implant-induced weight gain or estrogen-induced thrombosis, allowing for truly personalized prescribing that bypasses the trial-and-error approach that currently frustrates so many patients [48].

### 8.3. The Role of Family Planning

A recent systematic review examined the relationship between family planning use and the nutritional status of adolescent girls and women of reproductive age. The findings suggest that improved access to family planning may positively influence nutritional outcomes by supporting healthier birth spacing and overall maternal health. The authors also highlight the need for further research to better clarify the direct nutritional impacts of family planning interventions [49].

The committee statement by Zwayne and colleagues reviews current evidence on the relationship between hormonal contraception and body weight, concluding that most hormonal contraceptive methods (e.g., combined oral contraceptives, progestin-only pills, implants, injections, and hormonal IUDs) are not associated with clinically significant weight gain. The authors emphasize the importance of evidence-based counseling to address misconceptions about weight changes. This approach supports informed decision-making and promotes effective family planning by ensuring that concerns about body weight do not unnecessarily limit contraceptive use [50].

## 9. Conclusions

The systematic/narrative review of the evidence leads to a conclusion that is neither a complete validation nor a complete dismissal of the weight gain concern.

1. The Myth: The belief that Combined Oral Contraceptives, the Patch, the Ring, and Hormonal IUDs cause accumulation of body fat is not supported by consistent high-quality evidence. High-quality, placebo-controlled evidence demonstrates these methods are weight-neutral. Observed changes are typically due to fluid retention (RAAS mediated) or secular trends in adult weight gain.
2. The Reality: With Depot Medroxyprogesterone Acetate (DMPA), weight gain is a reality. It poses a genuine risk of significant fat accumulation via hypothalamic appetite stimulation and glucocorticoid effects, particularly in adolescents.
3. The Outlier: The Contraceptive Implant occupies a middle ground. While weight-neutral for the population mean, it carries a very real risk of significant weight gain for a genetically distinct sub-population (ESR1 variant carriers).
4. The Mechanism: The distinction between fluid (estrogen/RAAS) and fat (progestin/appetite) is critical. Clinicians must educate patients on this difference to manage expectations and side effects effectively.

Ultimately, the myth of weight gain is a narrative that persists because it is partially grounded in the reality of fluid shifts and the genuine metabolic effects of high-dose injectables, then generalized to all methods. Deconstructing this myth requires a commitment to precision medicine—acknowledging that while the average woman does not gain weight, the individual woman in the exam room might be the genetic exception who does.

## Figures and Tables

**Table 1:** Comparative Body Composition Changes (Fat vs. Lean Mass).

Comparison	Findings
DMPA vs. Copper IUD	DMPA users gained +4.1 kg fat mass; IUD users gained minimal weight. DMPA users had increased central/visceral fat.
LNG-IUS vs. Copper IUD	No difference in fat mass. Both groups gained small amount of lean body mass.
Implant vs. Copper IUD	No significant difference in fat mass in adjusted models.
Patch vs. Baseline	Increase in Total Body Water, no significant increase in fat mass.

DMPA—Depot Medroxyprogesterone Acetate; IUD—intrauterine device; LNG-IUS—Levonorgestrel-releasing Intrauterine System.

**Table 2:** Comparative Weight Outcomes in Combined Hormonal Contraceptives.

Method	Hormone Composition	Mean Weight Change (6–12 mos.)	Primary Mechanism of Change	Evidence Quality
COC (LNG)	Ethinyl Estradiol + LNG	+0.5 kg (non-sig vs. placebo)	Fluid retention (RAAS)	High (Cochrane)
COC (DRSP)	Ethinyl Estradiol + DRSP	−0.5 to 0 kg	Diuretic effect (Anti-mineralocorticoid)	High (RCTs)
Transdermal Patch	Ethinyl Estradiol + Norelgestromin	+0.6 kg	Interstitial fluid hydration	Moderate (Cohort)
Vaginal Ring	Ethinyl Estradiol + Etonogestrel	Neutral	Minimal systemic estrogen load	Moderate (Comparative)

LNG—Levonorgestrel; DRSP—Drospirenone; non-sig—non-significant; RAAS—Renin–Angiotensin–Aldosterone System; RCTs—Randomized Controlled Trials.

**Table 3:** Weight Gain Risk Stratified by Contraceptive Method.

Method	Hormone Type	Est. 12-Month Weight Change	Risk of Significant Gain (>2 kg)	Primary Mechanism
Combined Pill (COC)	Estrogen + Progestin	Neutral (+/−0.5 kg)	Low	Fluid Retention (RAAS)
Transdermal Patch	Estrogen + Progestin	Neutral (+0.6 kg)	Low	Interstitial Fluid Shift
Vaginal Ring	Estrogen + Progestin	Neutral	Low	N/A
Progestin-Only Pill	Progestin	Neutral (<2 kg)	Low	Minimal Systemic Effect
Hormonal IUD (LNG)	Levonorgestrel	Neutral (+0.5 kg)	Low	Local Uterine Effect
Implant (Nexplanon)	Etonogestrel	Variable (Avg +0.1–3 kg)	Moderate (Genetic dependent)	ESR1 Variant Interaction
Depo-Provera (DMPA)	Medroxyprogesterone	Gain (+2 to +6 kg)	High	Hypothalamic Appetite Stimulation

COC—combined oral contraceptive; IUD—intrauterine device; LNG—levonorgestrel; DMPA—Depot Medroxyprogesterone Acetate; Est.—estimated; RAAS—Renin–Angiotensin–Aldosterone System; N/A—Not Applicable; ESR1—Estrogen Receptor 1.

## Data Availability

No new data were created or analyzed in this study. Data sharing is not applicable to this article.
